# Ablation for Atrial Fibrillation: Can We Individualise Therapy or Should One Size Fit All?

**DOI:** 10.31083/RCM43927

**Published:** 2025-12-22

**Authors:** Nick B Spath, Ruairidh Martin, Moloy Das, Hanney Gonna, Prash Sanders, Kadhim Kadhim

**Affiliations:** ^1^Department of Cardiology, Edinburgh Royal Infirmary, EH16 4SA Edinburgh, UK; ^2^Department of Cardiology, Freeman Hospital, NE7 7DN Newcastle upon-Tyne, UK; ^3^Centre for Heart Rhythm Disorders, University of Adelaide and Royal Adelaide Hospital, Adelaide, SA 5000, Australia

**Keywords:** catheter ablation, atrial fibrillation, pulsed field ablation, spatiotemporal dispersion, vein of Marshall ethanol ablation

## Abstract

Modern medicine increasingly offers the potential to individualise patient care and tailor therapies to meet specific patient needs. Catheter ablation in atrial fibrillation has undergone radical evolution since the advent of early ablative therapies; however, more comprehensive or extensive strategies are now possible. Moreover, novel energy sources, catheters, and mapping platforms are being developed and implemented, raising the potential to deliver ablation strategies more effectively, durably, quickly, and potentially more extensively. This poses the challenge of whether to prioritise anatomical landmark-based ablation or pursue individual mechanisms of arrhythmia on a personalised basis. Thus, this review aims to summarise the current state-of-the-art developments in catheter ablation for atrial fibrillation, recent advances, and developments in both the ablation and understanding of arrhythmia pathophysiology.

## 1. Introduction

Atrial fibrillation (AF) is the most common cardiac arrhythmia worldwide and has substantial impact on morbidity and mortality, as well as significant associated healthcare costs to both primary and secondary care [1]. With increasing longevity and the rise of key risk factors, the prevalence of AF continues to rise to increase towards epidemic proportions and is set to double by the year 2050 [2, 3].

As the importance of early rhythm control has become recognised, catheter ablation for symptomatic AF has become the cornerstone of management reducing AF recurrence, burden and progression, as well as improving quality of life indices over and above treatment with anti-arrhythmic drugs [4, 5, 6, 7, 8]. Consequently, catheter ablation is given a Class I recommendation as a first-line therapy in patients with paroxysmal AF, with a Class IIb recommendation for selected patients with persistent AF [9] even outside the context of patients with heart failure in whom catheter ablation is arguably of most importance [7, 10].

As the field of catheter ablation in AF has evolved, we have seen a shifting dynamic between ablation strategies which target anatomical landmarks alone irrespective of other factors and those which seek to identify patient-specific substrate within the atria as targets for ablation. The formative early work demonstrating the importance of pulmonary vein triggers has led to pulmonary vein isolation (PVI) being the foundation of AF ablation [11] but it remains unclear what, if any, ablation therapies may be beneficial beyond this and how best to identify which strategies benefit which patients.

Whilst PVI without adjunctive ablation strategies has been shown to benefit a large proportion of patients, there remains a substantial group of patients in whom AF recurs despite successful isolation of the pulmonary veins [12]. This becomes more challenging still in patients with persistent and long-standing AF [13]. Therefore, the goal of achieving and maintaining sinus rhythm requires not only improving the delivery of successful and durable PVI, but also an improved understanding of the pathophysiology of AF to allow elimination of non-pulmonary vein triggers and substrate modification, when applicable. Multiple adjunctive ablation strategies, beyond PVI, have shown inconsistent results with poor reproducibility, further solidifying the importance of PVI as the cornerstone of AF ablation [14].

Since its inception, catheter ablation for AF has witnessed multiple evolutions aiming to achieve durable lesion creation, safely, and efficiently. Point-by-point radiofrequency (RF) ablation in particular, has had multiple advancement in terms of technology, including tip-irrigation, contact-force sensing, and refined delivery parameters integrated into 3D mapping systems enabling catheter stability monitoring and contiguous lesion delivery [15]. First-time AF ablations commonly utilise single-shot modalities designed to achieve PVI. Cryoballoon ablation platforms offer a short procedure duration with excellent safety profiles and have may have a shorter learning curve for operators but are less adaptable to anatomical variants. Two landmark randomised controlled trials assessing efficacy between single-shot cryoballoon and point-by-point radiofrequency PVI found no difference in clinical or safety endpoints [16, 17] although CIRCA-DOSE found a higher rate of progression to persistent atrial arrhythmia in patients undergoing cryoballoon therapy compared to radiofrequency ablation [18]. Despite very high acute procedural success rates, longer-term freedom from atrial arrhythmia with cryoballoon PVI is consistently less than 80% [19] with problematic reconnections in the carinas [20], which drives the search for other time-efficient approaches with higher clinical efficacy.

Pulsed-field ablation has come to the fore as an alternative energy modality for both single-shot PVI and focal applications. An adaptation of early ablations which utilised direct current, pulsed-field ablation causes both reversible and irreversible electroporation of cardiac cell membranes and offers the potential for higher success rates and favourable procedure duration and safety profiles, with some promise of added safety through cardioselectivity and sparing of surrounding structures.

This has resulted in an exciting new era in catheter ablation in AF, with new energy delivery systems and catheters as well as novel ways to enhance our understanding of pathophysiology of AF. This review focusses on the unmet needs in current catheter therapies for AF, novel developments to address these and standardised versus individualised ablation strategies.

## 2. Current Standards of Care, Challenges & Unmet Needs

### 2.1 Risk Factors, Indications & Contra-Indications to Ablation

Fundamental to aligning the most appropriate therapy to those who will derive the most benefit is patient selection. Management of risk factors is crucial to the wider management of AF and this is central to the principles of AF-CARE as compiled in recent guidelines [9]. Without adequately addressing hypertension, heart failure, obesity, sleep apnoea, alcohol and diabetes, many argue that proceeding to catheter ablation is not justified as the chances of long-term success are significantly reduced and the risk-benefit calculation around the procedure is therefore substantially different.

The LEGACY trial enrolled patients and offered weight and risk-factor management, observing a 6-fold increase in arrhythmia-free survival at 5-year follow-up in patients who lost ≥10% body weight, compared to patients who achieved less marked weight loss [21]. Of these patients with ≥10% weight loss, reversal from persistent to paroxysmal or no AF was observed in 88% at follow up, in contrast to the cohort achieving the least weight loss in whom 80% had no change or regressed from persistent to paroxysmal AF [22]. Physical activity and improved cardiorespiratory fitness have been shown to be associated with reduced arrhythmia recurrence and improved AF-related symptom burden, irrespective of weight loss as initially observed in the CARDIO-FIT study [23], and later prospectively tested in the ACTIVE-AF randomised controlled trial [24]. Emphasising the importance of addressing the modifiable risk factors holistically, beyond simply weight management, was the ARREST-AF cohort study. Aggressive risk factor management including weight loss, enhanced blood pressure and glycaemic control, in addition to identifying and treating sleep-disordered breathing when present, resulted in a near five-fold increase in the chances of maintaining sinus rhythm after multiple ablations (multivariable hazard ratio for risk factor modification = 4.8, 95% confidence interval: 2.04–11.4, *p* < 0.001) [25]. Further, a comprehensive risk factor modification approach has been shown to be associated with reversal of AF type as reported in the REVERSE-AF study, which demonstrated that nearly 90% of patients with persistent AF who achieved ≥10% weight loss, their AF regressed to paroxysmal underpinning the role of risk factor modification in AF management [22]. Subsequently, risk factor modification has been recognised in multiple societal guidelines and scientific statements [26].

Research and development of novel mapping and ablation modalities has endeavoured to characterise triggers and substrate underlying AF in the hope that these can be directly addressed with catheter intervention. Low-voltage areas have been repeatedly implicated in pathogenesis of AF, however they do not appear to be independently effective therapeutic targets [27, 28]. Fractionation of atrial electrograms [29, 30] as well as pattern and waveform [31, 32] are also recognised as relevant contributors to AF-triggering but have yet to be proven to benefit all-comers as part of an upfront ablation strategy. Overall, these data remind us how closely risk factors drive substrate and underscore the importance of risk factor management in any treatment strategy for AF [33].

Beyond these risk factors, patients who benefit most from catheter ablation therapies are those with symptomatic high-burden AF resistant to anti-arrhythmic drug therapy as well as those with cardiac failure [7, 34, 35] and these groups should therefore be prioritised for catheter-based strategies.

### 2.2 Importance of Time to Therapy 

Time from diagnosis to ablation is increasingly recognised as an important metric, with improved AF burden, recurrence, progression and symptom control [36], as well as potential for longer-term reduction in stroke risk and impact on cardiac function. Practices around catheter ablation as a first-line rhythm control strategy vary between centres and between countries. Multiple randomised studies have shown reduced AF burden as well as improvement of symptoms at 12–24 months with first-line ablative strategies particularly for symptomatic paroxysmal AF [37, 38, 39] without significantly higher adverse events. Analyses of healthcare costs comparing early ablation strategies to anti-arrhythmic drug therapies have consistently predicted cost-savings, predominantly driven by quality-adjusted life years gained [40, 41]. Whilst there are data to suggest that waiting up to 1 year for catheter ablation whilst on anti-arrhythmic drugs can be reasonable [42], it is increasingly recognised therefore that early ablative strategies can bring benefit for both patients and healthcare systems.

### 2.3 Radiofrequency Pulmonary Vein Isolation: Wide Area Circumferential Ablation

Radiofrequency PVI has historically been the backbone of ablation for AF. As electroanatomical mapping platforms have evolved with very high degrees of spatial and temporal resolution, ablation protocols have been standardised to deliver effective PVI reproducibly and safely. To mitigate against extra-cardiac thermal injury, alternative thresholds for energy delivery are applied to vulnerable areas such as the posterior wall. These protocols have been shown to improve safety whilst delivering effective lesions in these areas.

Ablation lesions in the left atrium are standardised by some mapping platforms (Lesion Size Index, Abbott; Ablation Index/Tag Index [43], CARTO, Johnson & Johnson Med Tech) whilst others use impedance only (Rhythmia, Boston Scientific, Marlborough, MA, USA [44]). Use of these standardised measures of lesion delivery with well-defined protocols have shown a high degree of acute procedural success [45]. The CLOSE-to-CURE study prospectively examined contact force-guided pulmonary vein isolation targeting an intertag distance of ≤6 mm and a region-specific ablation index (400 posterior wall, 550 elsewhere) demonstrated a significant reduction in arrhythmia burden, with 87% freedom from arrhythmia at 1 year, and 78% at 2 years [46].

### 2.4 Single-Shot: Cryoballoon Pulmonary Vein Isolation

The advent of cryoballoon PVI represented an innovative method to deliver acutely effective therapy to most patients as an alternative strategy. It offered comparable rates of venous isolation to radiofrequency, without compromising procedure time or safety. Furthermore, cryoballoon PVI is achievable in most patients without deep sedation or general anaesthesia, improving efficiency of workflow and service delivery, particularly in lower-volume centres.

The main challenge with cryoballoon platforms is variant anatomy, in particular common venous anatomy, where adaptability suffers in comparison to radiofrequency point-by-point approaches. Consequently, pre-procedural cross-sectional imaging is performed routinely in some healthcare systems [47, 48]. Where reconnection occurs, it is more commonly observed in the right-sided veins, although common venous anatomy also provides a challenge [49].

### 2.5 Challenges and Unmet Needs

Intraprocedural patient comfort and stability is of paramount importance in delivering effective ablation. In addition to the suffering caused by pain, inadequate sedation causes patients to move, increasing risk and jeopardising the accuracy of electroanatomical mapping platforms. Equally, deeper levels of conscious sedation can lead to erratic respiration patterns, especially in patients with obstructive sleep apnoea, resulting in greater catheter instability. Deep sedation protocols using propofol or general anaesthesia are highly effective in overcoming these challenges but access to such resources is far from widespread. Efforts to improve access to these by adopting and formalising physician-directed propofol have been well-received in some settings but require direct involvement of anaesthetists in others [50, 51].

High rates of recurrence of atrial arrhythmia have long been the Achilles heel of catheter ablation for AF. This is a greater challenge in patients with persistent AF and with dilated atria but even in paroxysmal AF whilst most series report very high success of acute PVI, atrial arrhythmia recurrence can be as high as 63% at 10 years [52]. This has driven the search for improved techniques in PVI but also in understanding factors above and beyond anatomical approaches.

There remains a lack of consensus on the most appropriate ablation strategy in a patient with recurrence of AF despite effective PVI in either paroxysmal or persistent AF. Additional ablation lines (roof lines, posterior wall isolation, mitral lines) in all patients during the index procedure has been shown to confer no additional benefit in terms of AF recurrence to PVI alone in persistent AF [53]. However, in repeat procedures with confirmed isolated pulmonary veins, most operators will consider additional lines based on empirical anatomical sites, the presence of scar in different regions of the left atrium, electrogram morphology or patterns, or other factors such as abnormal activity in the superior vena cava, highlighting the importance of techniques to individualise ablation therapies in some cases.

## 3. Recent Advances in AF Ablation

Evolution of ultra high density mapping catheters has significant advanced speed and accuracy of map generation for left atrial ablation for AF and has proven a useful tool in understanding the mechanisms of reconnection in cases of reconnection [54]. As a result of greater understanding of mechanisms of failure of PVI has driven advances in techniques to deliver more durable ablation.

### 3.1 High-Power Short-Duration Radiofrequency Ablation

Whilst more versatile than cryoballoon PVI in patients with large veins, dilated atria, and common venous anatomy, radiofrequency point-by-point PVI requires good catheter stability for accurate and durable lesion delivery. This challenge is increased further without general anaesthesia due to patient discomfort and erratic respiratory patterns. Lower power lesion delivery for greater time can paradoxically increase lesion depth through conductive heating, increasing risk of damage to extra-cardiac structures such as the oesophagus [55], a risk which garners considerable anxiety despite being an extremely rare complication [56].

High-power short-duration ablation protocols offer potential to limit conductive tissue damage and control lesion depth in higher risk areas such as the posterior wall. A recent meta-analysis of 6 randomised controlled trials found significant shorter procedure duration and ablation times with high-power short-duration strategies compared to a conventional approach [57]. Very-high-power short-duration strategies take this further delivering up to 90 Watts for up to 4 seconds, whereby depth of ablation lesions are reduced due to less thermal latency [58]. High- and very-high-power short-duration temperature-controlled ablation lesions produce lesions with greater diameter but less depth [59, 60]. Shorter lesion delivery protocols are very attractive in healthcare systems with more limited access to general anaesthesia and deep sedation, however clinical efficacy is key and in a recent study with high-power short-duration ablation was unable to match cryoballoon PVI [61].

### 3.2 Ethanol Ablation of the Vein of Marshall 

Ethanol ablation of the vein of Marshall targets areas of potentially key arrhythmogenic substrate for AF as well as peri-mitral flutter [62, 63]. Retrograde cannulation and occlusive infusion of ethanol causes effective upstream tissue necrosis and denervation which may have a beneficial impact on initiation and maintenance of AF [64].

Several observational studies and a single randomised controlled trial of ethanol ablation of the vein of Marshall have consistently reported reduced rates of recurrence of AF and atrial tachycardias beyond PVI alone [65]. However, recurrence rates remain significant with less than 70% freedom from AF or atrial tachycardia at 36 months [66], indicating a substantial proportion of patients for whom this adjunctive therapy is ineffective. Rates of successful mitral isthmus block are substantially higher in the Marshall vein ethanol ablation groups suggesting this form of ablation may be important for patients with peri-mitral flutter or focal atrial tachycardia arising from the mitral isthmus [65]. Recent data also suggest ethanol ablation of the Marshall vein remains important in achieving mitral isthmus block despite use of novel pulsed field ablation techniques [67].

One potential factor attenuating impact on AF from vein of Marshall ethanol ablation in published data is the rate of success for identifying, cannulating and ablating the vein of Marshall, which is not possible in every patient. Success rates range from 84% to 91% [68, 69] and further randomised trial data are needed. At present, the evidence does not support routine adoption of ethanol ablation of the vein of Marshall as part of routine workflow for catheter ablation of AF, but it remains an important treatment in selected patients.

### 3.3 Pulsed Field Ablation

Recent years have seen a dramatic expansion in the research and use of pulsed field ablation for AF ablation with multiple catheters in use or development at present. With its origins in direct current ablation first used for atrioventricular node ablation in humans in 1982 [70], pulsed field ablation applies a more refined dose and waveform of direct current to induce both reversible and irreversible electroporation of myocardial cells but with the promise of improved tissue-selectivity and safety profile over the primarily thermal ablation delivered by cryoballoon and RF ablation [70].

There have been multiple clinical studies to date with catheters designed for single-shot PVI with pulsed field ablation (Farapulse, Boston Scientific, Marlborough, MA, USA; PulseSelect, Medtronic, Minneapolis, MN, USA; Varipulse, Johnson & Johnson MedTech, New Brunswick, NJ, USA; Volt, Abbott, IL, Chicago, USA). The largest of these trials, ADVENT, assessed a pentaspline catheter (Farapulse, Boston Scientific) in patients with paroxysmal AF and was industry sponsored. ADVENT recruited over 300 patients to each of two arms receiving direct thermal (cryoballoon/radiofrequency) or pulsed field ablation [71]. The study showed non-inferiority with no difference between patients meeting the primary endpoint of recurrence of atrial arrhythmia at 12 months. MANIFEST-PF reported a large registry, incorporating 1568 patients from 24 European centres, of patients with paroxysmal or persistent AF treated with pulsed field ablation for PVI [72]. The freedom from atrial arrhythmia at median follow-up of 1 year was 78%, with adverse events reported in 1.9%. Overall, single-shot pulsed field ablation devices have shown non-inferiority to current modalities, with trends to shorter procedure duration [73] and with no clear signal of increased adverse risk to patients [74].

To date there has been only one investigator-initiated randomised controlled trial [73] which undertook a multi-centre head-to-head comparison of cryoballoon and pulsed field ablation PVI. With a non-inferiority design, 105 patients were recruited 1:1 to each modality with a primary endpoint of first recurrence of atrial arrhythmia at 3–12 months with a non-inferiority margin of 20%. Pulse field ablation reached non-inferiority with a signal for a lower rate of atrial arrhythmia recurrence at 12 months. Some key features of this important study warrant discussion. The rate of recurrence in both groups was higher than has been reported in other series. The high incidence of reported common venous anatomy in the cryoballoon group (20% versus 7%) may be partly explanatory. The higher reported recurrence rates than other published studies may also be partially explained by the definition of ≥30 s of atrial arrhythmia on implantable loop recorder, irrespective of symptoms. The authors reported that two patients in the pulsed field ablation cohort presented acutely to the emergency department with atrial arrhythmia, compared to 8 in the cryoballoon group. Importantly, the confidence interval for this observation was very wide and should be interpreted with caution in a study that was powered for non-inferiority.

Focal pulsed field ablation will soon be integrated with all industry mapping platforms but is already independently available for use with current commercial systems (CENTAURI™, CardioFocus). Designed to work across platforms with existing irrigated radiofrequency catheters, this open-system third party generator offers point-by-point lesion creation with delivery times of 4–6 s. The multicentre ECLIPSE AF trial enrolled 82 patients to point-by-point PVI with focal pulsed field ablation with all commercially available mapping and ablation platforms, looking at acute and 3-month isolation along with safety endpoints [75]. The investigators optimised the ablation strategy following analysis of the first 2 cohorts and reported per-PVI rate of 89% at 3 months in optimised cohorts. There was no signal for harm relating to pulsed field ablation therapy but the study was not powered for safety endpoints. Further studies are needed to assess longer term efficacy but there is real potential for focal pulsed field ablation to have wider applicability and utility across the whole field of invasive electrophysiology, with applications in both atria and ventricles. Beyond PVI, prospective data have also shown promise of this focal pulsed field ablation system for cavotricuspid isthmus ablation as well as non-PVI left atrial lesion sets, with a high rate of first-pass block [76].

There are several different designs of catheter and waveform of pulsed field ablation in clinical use and development, varying by manufacturer. Individual parameters of delivered pulsed field ablation energy consist of the amplitude, polarity, pulse duration, number of pulses and interpulse delay which collectively determine the effect on local tissues as well as safety profiles [77]. Pulse sequences are individual to specific platforms and not presently amenable to being changed by the operator. The different core treatment parameters of different pulsed field ablation products may have varying effects on lesion size and efficacy, which could in turn impact reversible electroporation and longer term recurrence of arrhythmia [78]. There have been safety concerns with specific platforms over increased risk of systemic thromboembolism requiring modification of their usage [79] and safety profiles are likely to differ between individual pulsed field ablation product. It is therefore crucial to adequately characterise acute and longer-term safety data prior to widespread adoption of these catheters to the market and replacement of other established modalities.

A key recognised advantage of pulsed field ablation is the reduction in risk of atrio-oesophageal fistula to the extent where it has never been reported in clinical application of the energy source [80] but other safety concerns specific to the modality have emerged. Acute arterial spasm has been reported with pulsed field ablation applications to areas of the heart in close proximity to coronary arteries, with longitudinal data suggesting that longer-term stenosis cannot be excluded in such cases [81]. Additional concerns have been raised about intravascular haemolysis, which occurs commonly in cases of very high doses of pulsed field ablation, but clinical impact is yet to be fully understood and widespread harm has not been evident from modest applications [82].

At the time of writing, there has been no pulsed field ablation system adopted in widespread clinical practice which can be used without general anaesthesia or deep sedation. This is largely due to skeletal muscle contraction and pain associated with the energy delivery. This poses a logistical challenge for many healthcare systems where routine access to anaesthetic support is variable. There remains lack of agreement on the relevance of marginal procedure time gain with pulsed field ablation modalities if overall catheter laboratory utilisation time is longer because of requirements for general anaesthesia.

Despite understandable enthusiasm for this novel ablation modality which has potential to revolutionise practice moving forwards, robust short-term randomised trials and long-term outcome and safety data are crucial before widespread adoption into mainstream practice.

## 4. Individualised Versus Anatomical Therapy

### 4.1 Initial Individualised Therapy: CFAE Ablation

**Fig. 1.**
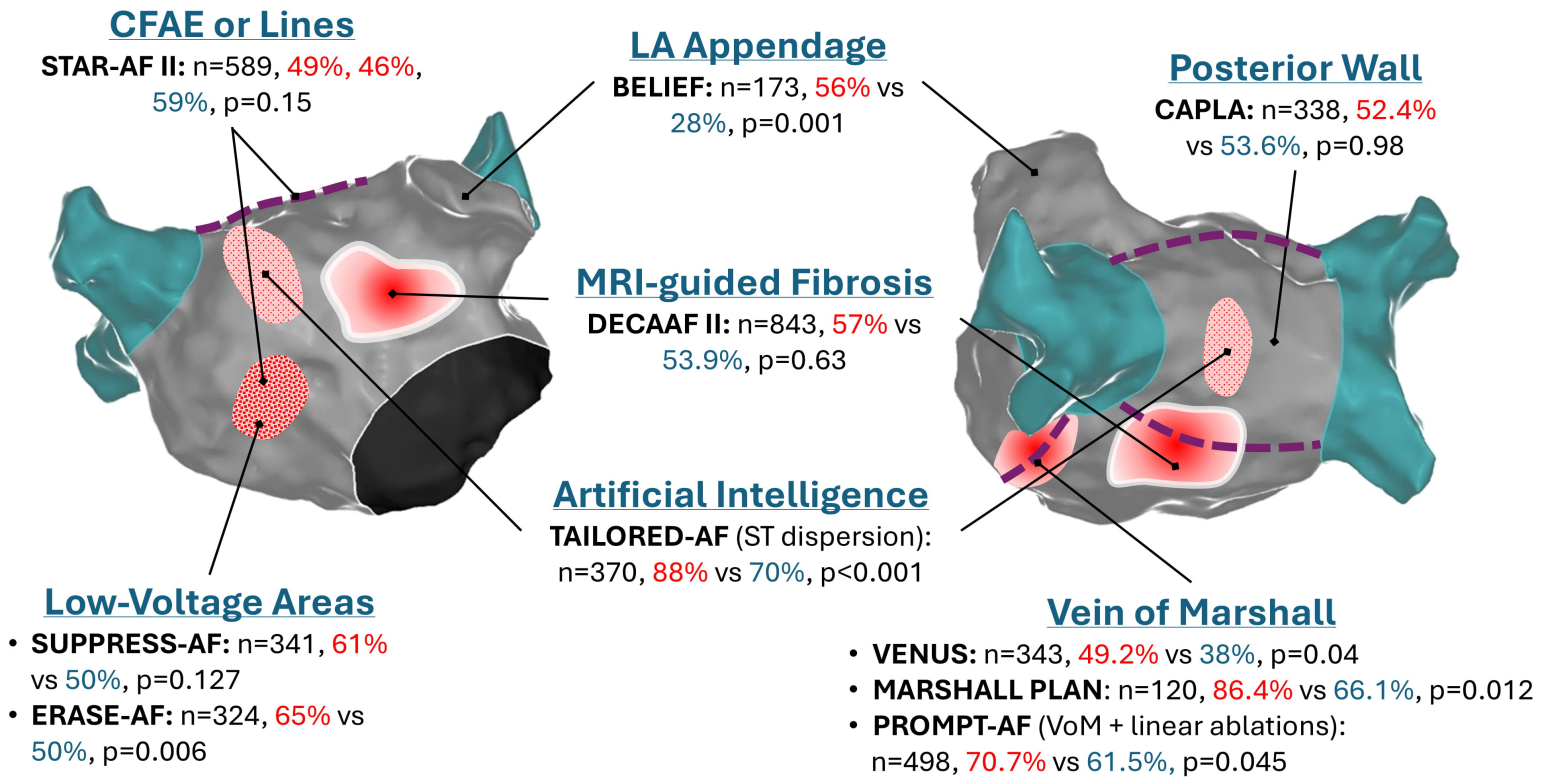
**Summary of notable randomised controlled trials of catheter ablation beyond PVI for persistent AF**. Red %: proportion of patients receiving interventional ablation who are free of arrhythmia at follow-up (typically 12 months). Blue %: proportion of arrhythmia-free patients receiving PVI alone. CFAE, complex fractionated electrograms; LA, left atrium; VoM, Vein of Marshall; PVI, pulmonary vein isolation; AF, atrial fibrillation; MRI, magnetic resonance imaging.

The origins of catheter ablation for AF were rooted in trying to identify specific causative triggers in the individual atrium undergoing treatment, first focussing on pulmonary vein triggers and evolving to target complex fractionated atrial electrograms for ablation [83]. Felt to be of particular relevance to patients with persistent AF in whom outcomes from PVI alone have been inferior [84], this approach is time-consuming and requires a high degree of operator skill and experience. STAR AF I looked exclusively at quality of life indices following PVI or additional ablation of complex fractionated atrial electrograms, finding no impact of the additional therapy on symptom burden up to 1 year [85]. In STAR AF II, patients were randomised to PVI alone, PVI plus complex fractionated electrogram ablation or PVI plus linear roof and mitral isthmus ablation [53]. Aside from significantly longer procedure times, there were no discernible differences between the groups, suggesting that upfront additional ablation beyond the pulmonary veins in all-comers is not a beneficial strategy. Fig. 1 summarises key findings from randomised controlled trials examining ablation approaches beyond PVI in patients with persistent AF.

### 4.2 Anatomical Approaches

In recent years, ablation strategies have moved from these more individualised efforts to a standardised anatomical approach. The cornerstone of this is isolation of pulmonary veins. There remains broad lack of consensus as to whether more ablation is indicated and if so, what form this additional therapy should take.

As an alternative addition to PVI alone, coronary sinus isolation was recently assessed in patients with high-burden (long-episode paroxysmal or persistent) AF [86]. Participants were randomised to either coronary sinus and PVI plus a roof line or PVI and a roof line only, but no additive value was seen with the addition of this ablation target. The CAPLA trial assessed the impact of additional posterior wall to PVI alone in patients with persistent AF, again reporting no additional benefit freedom from arrhythmia at 1 year follow-up [87] (Fig. 1). STAR-AF III intends to assess the specific additive value over and above PVI of posterior wall isolation or ablation of AF drivers using contemporary catheter technologies, with results anticipated in 2027 [88]. Targeting the left atrial appendage as an arrhythmogenic structure is an interesting hypothesis, that was tested in the BELIEF trial [89]. Empirical left atrial appendage isolation, added to extensive left atrial ablation, resulted in a near-doubling of arrhythmia-free survival in a small cohort of longstanding persistent AF patients (n = 173, unadjusted hazard ratio [HR] for recurrence with standard ablation: 1.92; 95% confidence interval [CI]: 1.3 to 2.9; log-rank *p* = 0.001). However, this approach has not been widely adopted due to concerns regarding thrombosis and stroke risk that are associated with reducing left atrial appendage mechanical emptying with electrical isolation.

The potential importance of left atrial fibrosis as substrate for arrhythmia has further driven substrate-based anatomical approaches to ablation, as magnetic resonance imaging-derived fibrosis extent independently predicts arrhythmia recurrence at 1 year following an index ablation procedure [90]. However, translating this to practice with ablation in individual regions of fibrosis in addition to PVI did not result in a difference in freedom from arrhythmia [91]. There was a signal for greater adverse events in the group receiving more extensive ablation, suggesting targeting anatomical image-defined substrate for arrhythmia in the left atrium is unlikely to be an appropriate upfront strategy in all patients.

Somewhat contrasting these data, the ERASE-AF trial randomised patients with persistent AF to PVI or additional low-voltage guided substrate ablation by multipolar mapping rather than imaging guidance, reporting a significantly lower recurrence rate at 1 year in the group receiving substrate-based ablation at 35% compared to 50% [92]. A trend to higher adverse events in the treatment arm did not reach statistical significance. More recently, Masuda and colleagues reported their findings from SUPPRESS-AF study, in which they randomised 341 patients with persistent AF and low-voltage areas (≥5 cm) after PVI to PVI alone, or PVI and low voltage ablation [93]. There was no difference in arrhythmia-free survival at 12 months (61% vs 50%, *p* = 0.127), with no difference in procedure-related complications.

Increasingly, there is recognition that it can be impossible in a proportion of patients to achieve durable bidirectional block with left atrial lines from endocardial radiofrequency ablation alone [94] indicating that epicardial connections are likely to play a role. Failure of durable ablation lines has been suggested as a contributing factor to higher recurrence rates. In light of this, groups have begun to explore hybrid endo-/epicardial ablation strategies in patients with persistent AF, with CEASE-AF employing a thoracoscopic epicardial ablation element and reporting dramatically improved freedom from arrhythmia at 1 year of 72% in the hybrid ablation arm compared to 39% with endocardial ablation alone [95]. EPIC-AF aims to address this question with a concomitant epicardial atrial catheter ablation arm to ensure bidirectional block is achieved in order to assess impact on outcomes [96]. Whether the same issues of endo-/epicardial transmurality will also apply to novel pulsed field ablation technologies remains to be seen.

The Marshall Plan proposes an extensive upfront approach with vein isolation as well as lines and ethanol ablation of the vein of Marshall [69]. Likewise, PROMPT-AF found that the addition of vein of Marshall ethanolisation and linear ablations (roof, mitral isthmus, and cavotricuspid isthmus) was associated with higher arrhythmia-free survival at 1 year (hazard ratio, 0.73; 95% CI, 0.54–0.99, *p* = 0.045) [97] (Fig. 1). Whilst early signs that lines with vein of Marshall ablation may confer modest benefit, the long-term data from the Marshall Plan are awaited and it is likely that multi-centre data will be required to know if there is true benefit across the population as a whole.

### 4.3 Spatiotemporal Dispersion Mapping

Artificial intelligence has most recently been applied to the field of catheter ablation for AF, in a rejuvenated effort to better understand mechanisms of arrhythmia and tailor therapies to individual patients. Focus has been returned to complex fractionated atrial electrograms but by using the power of artificial intelligence to assimilate large datasets of electrograms, analysing them for patterns of spatiotemporal dispersion to identify regions within the atrium which are more likely to be actively involved in triggering AF for the patient.

There are currently two platforms adopting artificial intelligence-assisted spatiotemporal dispersion with published data, one of which is available commercially (Volta AF-Xplorer™, Volta Medical, USA). This identifies three or more consecutive atrial electrograms which occupy the majority of the AF cycle length, highlighting these areas as potential key triggers. Ockham, usable as a research tool with Rhythmia™ (Boston Scientific) utilises an alternative artificial intelligence algorithm, comparing local electrograms according to cycle length and spread of activation within this cycle length, incorporating stability, providing a ranking of key regions of rapid but stable fractionation relative to other areas [98]. Both methods share the aim of giving a tailored understanding of the pathophysiological mechanisms of arrhythmia in an individual patient basis, enabling therapy to be optimised.

One randomised control trial exists in this space to date, randomising 374 patients with persistent AF 1:1 to PVI alone using radiofrequency ablation or a tailored approach with additional spatiotemporal distribution-guided ablation [99]. Following a 3-month blanking period, freedom from AF was significantly higher in the group undergoing tailored therapy (88%) compared to standard therapy (70%). This difference was lost when considering freedom from all atrial arrhythmias but this was due to recurrence of left atrial macro re-entrant tachycardias which is perhaps unsurprising. Procedural and total ablation time were also longer in the tailored group but there were no differences in safety endpoints. This high rate of freedom from AF in a persistent population is striking and offers promise that understanding the specific mechanisms of arrhythmia in patients undergoing catheter ablation may be key to improving longer-term procedural success beyond purely anatomical approaches.

## 5. Conclusions & Future Directions

Electrophysiologists continually strive for the best outcomes in a population of increasingly co-morbid and complex patients. Despite advances in ablation catheters which have enabled more durable and effective lesion delivery, mapping platforms and novel energy delivery, there remains a significant portion of patients who return with recurrent AF within 12 months.

**Fig. 2.**
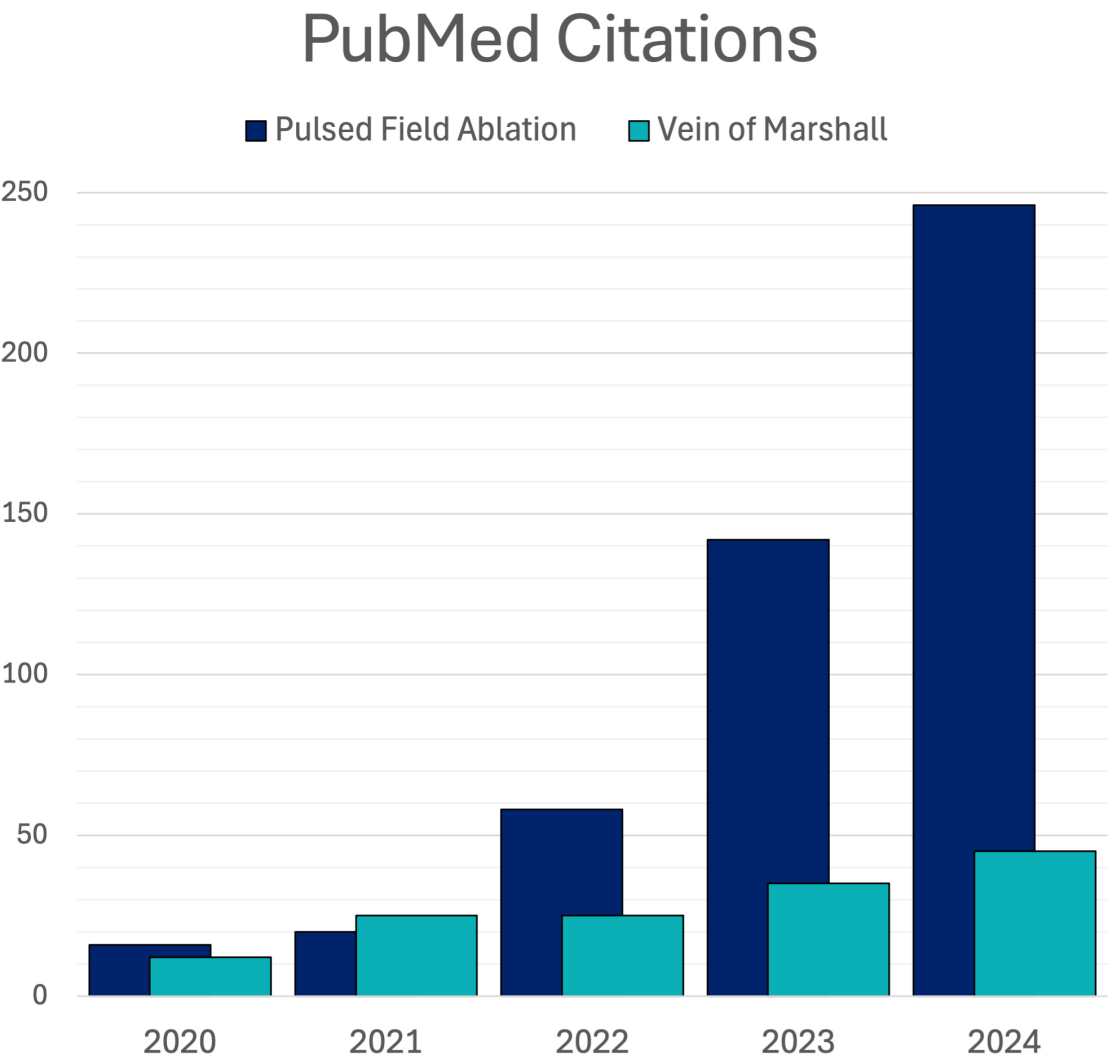
**PubMed Citations; Pulsed Field Ablation vs Vein of Marshall Ethanol Ablation**.

Pulsed field ablation has expanded rapidly (Fig. 2) and vigorous research continues to further understand and hone this novel ablation modality for both single-shot PVI and focal applications. The evidence to date presents an ablation workflow which appears safe and effective, with potential to have improved longer-term outcomes compared to existing techniques, though this is yet to be confirmed. Future studies are needed to evaluate efficacy of anatomical approaches in addition to PVI using pulsed-field ablation. Whether barriers around access to general anaesthesia will be overcome remains to be seen.

Can we individualise therapy in ablation for AF or should one size fit all? A wealth of published trial data tell us that anatomical approaches with additional ablation beyond PVI are, so far, yet to demonstrate significant impact on the population of patients with AF as a whole, although in selected patients they certainly have a role. It seems that we cannot, at present, remove expert experience and skill from informing what anatomical ablations will benefit individual patients. Early evidence is emerging that improved identification of individual triggers for AF may enhance our ability to achieve freedom from AF longer-term, harnessing the power of artificial intelligence to assimilate very large datasets. There remain key challenges in this area before it is clear that this approach will benefit the wider patient population as a whole and further work in the field of individualisation of ablation is needed to better understand its potential impact.

Whilst the many innovative advances in the field now mean there is a broad array of interventional treatment strategies for patients with AF, to pursue these in all patients blindly without first aggressively managing risk factors exposes patients to greater risk and reduces long-term procedural success rates, and therefore should be avoided. The modern-day electrophysiologist must use all tools at their disposal, both as an operator and a physician, in an effort to improve outcomes in what is a rapidly evolving and exciting era in the speciality.

## References

[1] Kornej J, Börschel CS, Benjamin EJ, Schnabel RB (2020). Epidemiology of Atrial Fibrillation in the 21st Century: Novel Methods and New Insights. *Circulation Research*.

[2] Linz D, Gawalko M, Betz K, Hendriks JM, Lip GYH, Vinter N (2023). Atrial fibrillation: epidemiology, screening and digital health. *The Lancet Regional Health*.

[3] Magnani S, Ali H, Cappato R (2025). Upgrade on atrial fibrillation ablation in the new ESC Guidelines. *European Heart Journal Supplements*.

[4] Calkins H, Reynolds MR, Spector P, Sondhi M, Xu Y, Martin A (2009). Treatment of atrial fibrillation with antiarrhythmic drugs or radiofrequency ablation: two systematic literature reviews and meta-analyses. *Circulation. Arrhythmia and Electrophysiology*.

[5] Packer DL, Kowal RC, Wheelan KR, Irwin JM, Champagne J, Guerra PG (2013). Cryoballoon ablation of pulmonary veins for paroxysmal atrial fibrillation: first results of the North American Arctic Front (STOP AF) pivotal trial. *Journal of the American College of Cardiology*.

[6] Mont L, Bisbal F, Hernández-Madrid A, Pérez-Castellano N, Viñolas X, Arenal A (2014). Catheter ablation vs. antiarrhythmic drug treatment of persistent atrial fibrillation: a multicentre, randomized, controlled trial (SARA study). *European Heart Journal*.

[7] Marrouche NF, Kheirkhahan M, Brachmann J (2018). Catheter Ablation for Atrial Fibrillation with Heart Failure. *The New England Journal of Medicine*.

[8] Dulai R, Sulke N, Freemantle N, Lambiase PD, Farwell D, Srinivasan NT (2024). Pulmonary Vein Isolation vs Sham Intervention in Symptomatic Atrial Fibrillation: The SHAM-PVI Randomized Clinical Trial. *JAMA*.

[9] Van Gelder IC, Rienstra M, Bunting KV, Casado-Arroyo R, Caso V, Crijns HJGM (2024). 2024 ESC Guidelines for the management of atrial fibrillation developed in collaboration with the European Association for Cardio-Thoracic Surgery (EACTS). *European Heart Journal*.

[10] Tzeis S, Gerstenfeld EP, Kalman J, Saad E, Shamloo AS, Andrade JG (2024). 2024 European Heart Rhythm Association/Heart Rhythm Society/Asia Pacific Heart Rhythm Society/Latin American Heart Rhythm Society expert consensus statement on catheter and surgical ablation of atrial fibrillation. *Journal of Interventional Cardiac Electrophysiology*.

[11] Haïssaguerre M, Jaïs P, Shah DC, Takahashi A, Hocini M, Quiniou G (1998). Spontaneous initiation of atrial fibrillation by ectopic beats originating in the pulmonary veins. *The New England Journal of Medicine*.

[12] Benali K, Barré V, Hermida A, Galand V, Milhem A, Philibert S (2023). Recurrences of Atrial Fibrillation Despite Durable Pulmonary Vein Isolation: The PARTY-PVI Study. *Circulation. Arrhythmia and Electrophysiology*.

[13] Calvert P, Ding WY, Griffin M, Bisson A, Koniari I, Fitzpatrick N (2024). Silent pulmonary veins at redo ablation for atrial fibrillation: Implications and approaches. *Journal of Interventional Cardiac Electrophysiology*.

[14] Johner N, Namdar M, Shah DC (2025). Catheter ablation of atrial fibrillation: time to look beyond iterative pulmonary vein isolation only and “one-size-fits-all” strategies. *Indian Pacing and Electrophysiology Journal*.

[15] De Pooter J, Strisciuglio T, El Haddad M, Wolf M, Phlips T, Vandekerckhove Y (2019). Pulmonary Vein Reconnection No Longer Occurs in the Majority of Patients After a Single Pulmonary Vein Isolation Procedure. *JACC. Clinical Electrophysiology*.

[16] Kuck KH, Brugada J, Fürnkranz A, Metzner A, Ouyang F, Chun KRJ (2016). Cryoballoon or Radiofrequency Ablation for Paroxysmal Atrial Fibrillation. *The New England Journal of Medicine*.

[17] Andrade JG, Champagne J, Dubuc M, Deyell MW, Verma A, Macle L (2019). Cryoballoon or Radiofrequency Ablation for Atrial Fibrillation Assessed by Continuous Monitoring: A Randomized Clinical Trial. *Circulation*.

[18] Andrade JG, Deyell MW, Khairy P, Champagne J, Leong-Sit P, Novak P (2024). Atrial fibrillation progression after cryoablation vs. radiofrequency ablation: the CIRCA-DOSE trial. *European Heart Journal*.

[19] Canpolat U, Kocyigit D, Yalcin MU, Coteli C, Sener YZ, Oksul M (2019). Long-term outcomes of pulmonary vein isolation using second-generation cryoballoon during atrial fibrillation ablation. *Pacing and Clinical Electrophysiology*.

[20] Gkalapis C, Vlachos K, Papadakis M, Pavleros N, Hippe HJ, Benali K (2025). Analysis of the effectiveness of the latest 4th-generation cryoballoon catheters in pulmonary vein isolation using high-resolution mapping. *Hellenic Journal of Cardiology*.

[21] Pathak RK, Middeldorp ME, Meredith M, Mehta AB, Mahajan R, Wong CX (2015). Long-Term Effect of Goal-Directed Weight Management in an Atrial Fibrillation Cohort: A Long-Term Follow-Up Study (LEGACY). *Journal of the American College of Cardiology*.

[22] Middeldorp ME, Pathak RK, Meredith M, Mehta AB, Elliott AD, Mahajan R (2018). PREVEntion and regReSsive Effect of weight-loss and risk factor modification on Atrial Fibrillation: the REVERSE-AF study. *Europace*.

[23] Pathak RK, Elliott A, Middeldorp ME, Meredith M, Mehta AB, Mahajan R (2015). Impact of CARDIOrespiratory FITness on Arrhythmia Recurrence in Obese Individuals With Atrial Fibrillation: The CARDIO-FIT Study. *Journal of the American College of Cardiology*.

[24] Elliott AD, Verdicchio CV, Mahajan R, Middeldorp ME, Gallagher C, Mishima RS (2023). An Exercise and Physical Activity Program in Patients With Atrial Fibrillation: The ACTIVE-AF Randomized Controlled Trial. *JACC. Clinical Electrophysiology*.

[25] Pathak RK, Middeldorp ME, Lau DH, Mehta AB, Mahajan R, Twomey D (2014). Aggressive risk factor reduction study for atrial fibrillation and implications for the outcome of ablation: the ARREST-AF cohort study. *Journal of the American College of Cardiology*.

[26] Chung MK, Eckhardt LL, Chen LY, Ahmed HM, Gopinathannair R, Joglar JA (2020). Lifestyle and Risk Factor Modification for Reduction of Atrial Fibrillation: A Scientific Statement From the American Heart Association. *Circulation*.

[27] Kaiser B, Huber C, Pirozzolo G, Maier P, Bekeredjian R, Theis C (2024). Persistent atrial fibrillation without the evidence of low-voltage areas: a prospective randomized trial. *Journal of Interventional Cardiac Electrophysiology*.

[28] Yagishita A, Sakama S, Iimura K, Lee KH, Ayabe K, Amino M (2025). Clinical relevance of left atrial structural remodeling and non-pulmonary vein foci in atrial fibrillation. *Journal of Interventional Cardiac Electrophysiology*.

[29] Rossi P, Cauti FM, Polselli M, Magnocavallo M, Niscola M, Fanti V (2024). Ablation of persistent atrial fibrillation based on atrial electrogram duration map: methodology and clinical outcomes from the AEDUM pilot study. *Journal of Interventional Cardiac Electrophysiology*.

[30] Mizobuchi M, Yamashita T, Sato T, Funatsu A, Kobayashi T, Nakamura S (2025). Selective complex fractionated atrial electrogram ablation based on the number-of-fractionation for persistent atrial fibrillation refractory to pulmonary vein isolation. *Journal of Interventional Cardiac Electrophysiology*.

[31] Riku S, Inden Y, Yanagisawa S, Fujii A, Tomomatsu T, Nakagomi T (2024). Distributions and number of drivers on real-time phase mapping associated with successful atrial fibrillation termination during catheter ablation for non-paroxysmal atrial fibrillation. *Journal of Interventional Cardiac Electrophysiology*.

[32] Sommer P, Castellano S, Ahapov K, Jansen MM, Mehta NK, Kong MH (2024). A single-center trial of electrographic flow mapping and concomitant voltage mapping in sinus rhythm and atrial fibrillation (FLOW EVAL-AF). *Journal of Interventional Cardiac Electrophysiology*.

[33] Kadhim K, Middeldorp ME, Elliott AD, Agbaedeng T, Gallagher C, Malik V (2021). Prevalence and Assessment of Sleep-Disordered Breathing in Patients With Atrial Fibrillation: A Systematic Review and Meta-analysis. *The Canadian Journal of Cardiology*.

[34] Jones DG, Haldar SK, Hussain W, Sharma R, Francis DP, Rahman-Haley SL (2013). A randomized trial to assess catheter ablation versus rate control in the management of persistent atrial fibrillation in heart failure. *Journal of the American College of Cardiology*.

[35] Fiala M, Wichterle D, Bulková V, Sknouril L, Nevralová R, Toman O (2014). A prospective evaluation of haemodynamics, functional status, and quality of life after radiofrequency catheter ablation of long-standing persistent atrial fibrillation. *Europace*.

[36] Andrade JG, Wells GA, Deyell MW, Bennett M, Essebag V, Champagne J (2021). Cryoablation or Drug Therapy for Initial Treatment of Atrial Fibrillation. *The New England Journal of Medicine*.

[37] Morillo CA, Verma A, Connolly SJ, Kuck KH, Nair GM, Champagne J (2014). Radiofrequency ablation vs antiarrhythmic drugs as first-line treatment of paroxysmal atrial fibrillation (RAAFT-2): a randomized trial. *JAMA*.

[38] Kuniss M, Pavlovic N, Velagic V, Hermida JS, Healey S, Arena G (2021). Cryoballoon ablation vs. antiarrhythmic drugs: first-line therapy for patients with paroxysmal atrial fibrillation. *Europace*.

[39] Razzack AA, Lak HM, Pothuru S, Rahman S, Hassan SA, Hussain N (2022). Efficacy and Safety of Catheter Ablation vs Antiarrhythmic Drugs as Initial Therapy for Management of Symptomatic Paroxysmal Atrial Fibrillation: A Meta-Analysis. *Reviews in Cardiovascular Medicine*.

[40] Andrade JG, Moss JWE, Kuniss M, Sadri H, Wazni O, Sale A (2024). The Cost-Effectiveness of First-Line Cryoablation vs First-Line Antiarrhythmic Drugs in Canadian Patients With Paroxysmal Atrial Fibrillation. *The Canadian Journal of Cardiology*.

[41] Wazni O, Moss J, Kuniss M, Andrade J, Chierchia GB, Mealing S (2023). An economic evaluation of first-line cryoballoon ablation vs antiarrhythmic drug therapy for the treatment of paroxysmal atrial fibrillation from a U.S. Medicare perspective. *Heart Rhythm O2*.

[42] Kalman JM, Al-Kaisey AM, Parameswaran R, Hawson J, Anderson RD, Lim M (2023). Impact of early vs. delayed atrial fibrillation catheter ablation on atrial arrhythmia recurrences. *European Heart Journal*.

[43] Das M, Loveday JJ, Wynn GJ, Gomes S, Saeed Y, Bonnett LJ (2017). Ablation index, a novel marker of ablation lesion quality: prediction of pulmonary vein reconnection at repeat electrophysiology study and regional differences in target values. *Europace*.

[44] García-Bolao I, Ramos P, Luik A, S Sulkin M, R Gutbrod S, Oesterlein T (2022). Local Impedance Drop Predicts Durable Conduction Block in Patients With Paroxysmal Atrial Fibrillation. *JACC. Clinical Electrophysiology*.

[45] Taghji P, El Haddad M, Phlips T, Wolf M, Knecht S, Vandekerckhove Y (2018). Evaluation of a Strategy Aiming to Enclose the Pulmonary Veins With Contiguous and Optimized Radiofrequency Lesions in Paroxysmal Atrial Fibrillation: A Pilot Study. *JACC. Clinical Electrophysiology*.

[46] Duytschaever M, De Pooter J, Demolder A, El Haddad M, Phlips T, Strisciuglio T (2020). Long-term impact of catheter ablation on arrhythmia burden in low-risk patients with paroxysmal atrial fibrillation: The CLOSE to CURE study. *Heart Rhythm*.

[47] Thorning C, Hamady M, Liaw JVP, Juli C, Lim PB, Dhawan R (2011). CT evaluation of pulmonary venous anatomy variation in patients undergoing catheter ablation for atrial fibrillation. *Clinical Imaging*.

[48] Merchant FM, Levy MR, Iravanian S, Clermont EC, Kelli HM, Eisner RL (2016). Pulmonary vein anatomy assessed by cardiac magnetic resonance imaging in patients undergoing initial atrial fibrillation ablation: implications for novel ablation technologies. *Journal of Interventional Cardiac Electrophysiology*.

[49] Obergassel J, Nies M, Kirchhof P, Metzner A (2024). Pulmonary vein reconnection and repeat ablation characteristics following cryoballoon- compared to radiofrequency-based pulmonary vein isolation. *Journal of Cardiovascular Electrophysiology*.

[50] Tilz R, Chun K, Deneke T, Kelm M, Piorkowski C, Sommer P (2017). Positionspapier der Deutschen Gesellschaft für Kardiologie zur Kardioanalgosedierung. *Kardiologe*.

[51] Tilz R, Busch S, Chun K, Frerker C, Gaede L, Steven D (2024). Analgosedierung in der Kardiologie Konsensuspapier der DGK und DGAI 2024. *Anasthesiologie & Intensivmedizin*.

[52] Tilz RR, Heeger CH, Wick A, Saguner AM, Metzner A, Rillig A (2018). Ten-Year Clinical Outcome After Circumferential Pulmonary Vein Isolation Utilizing the Hamburg Approach in Patients With Symptomatic Drug-Refractory Paroxysmal Atrial Fibrillation. *Circulation. Arrhythmia and Electrophysiology*.

[53] Verma A, Jiang CY, Betts TR, Chen J, Deisenhofer I, Mantovan R (2015). Approaches to catheter ablation for persistent atrial fibrillation. *The New England Journal of Medicine*.

[54] Gunawardene MA, Eickholt C, Akbulak RÖ, Jularic M, Klatt N, Hartmann J (2020). Ultra-high-density mapping of conduction gaps and atrial tachycardias: Distinctive patterns following pulmonary vein isolation with cryoballoon or contact-force-guided radiofrequency current. *Journal of Cardiovascular Electrophysiology*.

[55] Borne RT, Sauer WH, Zipse MM, Zheng L, Tzou W, Nguyen DT (2018). Longer Duration Versus Increasing Power During Radiofrequency Ablation Yields Different Ablation Lesion Characteristics. *JACC. Clinical Electrophysiology*.

[56] Lim MW, Kalman JM (2023). Putting fear into perspective: estimating the true incidence of oesophageal fistula formation post-atrial fibrillation ablation. *European Heart Journal*.

[57] Farnir FIP, Luermans JGLM, Farnir FPFJDJ, Chaldoupi SM, Linz D (2024). Impedance drop during focal monopolar pulsed field ablation in the atrium. *Journal of Interventional Cardiac Electrophysiology*.

[58] Teumer Y, Ziemssen H, Katov L, Bothner C, Mayer B, Rottbauer W (2025). Comparative lesion metrics analysis of very high power and high power short duration radiofrequency ablation in a Porcine ex vivo model. *Scientific Reports*.

[59] Reddy VY, Grimaldi M, De Potter T, Vijgen JM, Bulava A, Duytschaever MF (2019). Pulmonary Vein Isolation With Very High Power, Short Duration, Temperature-Controlled Lesions: The QDOT-FAST Trial. *JACC. Clinical Electrophysiology*.

[60] Heeger CH, Almorad A, Scherr D, Szegedi N, Seidl S, Baran J (2025). Temperature-guided high and very high-power short duration ablation for atrial fibrillation treatment: the peQasus multicentre study. *Europace*.

[61] Sultan A, Kreutzer S, Wörmann J, Lüker J, Ackmann J, Schipper JH (2025). HIgh Power short duration radiofrequency ablation or cryoballoon ablation for paroxysmal Atrial Fibrillation (HIPAF trial). *Europace*.

[62] Lee SH, Tai CT, Hsieh MH, Tsao HM, Lin YJ, Chang SL (2005). Predictors of non-pulmonary vein ectopic beats initiating paroxysmal atrial fibrillation: implication for catheter ablation. *Journal of the American College of Cardiology*.

[63] Kim DT, Lai AC, Hwang C, Fan LT, Karagueuzian HS, Chen PS (2000). The ligament of Marshall: a structural analysis in human hearts with implications for atrial arrhythmias. *Journal of the American College of Cardiology*.

[64] Valderrábano M, Chen HR, Sidhu J, Rao L, Ling Y, Khoury DS (2009). Retrograde ethanol infusion in the vein of Marshall: regional left atrial ablation, vagal denervation and feasibility in humans. *Circulation. Arrhythmia and Electrophysiology*.

[65] Ge WL, Li T, Lu YF, Jiang JJ, Tung TH, Yan SH (2024). Efficacy and feasibility of vein of Marshall ethanol infusion during persistent atrial fibrillation ablation: A systematic review and meta-analysis. *Clinical Cardiology*.

[66] Luo B, Ma Z, Liu X, Liu T, Guo X, Sun Q (2025). Long-term effects of catheter ablation with vein of Marshall ethanol infusion vs. pulmonary vein isolation alone on persistent atrial fibrillation. *International Journal of Cardiology*.

[67] Cubberley A, Ahmadian-Tehrani AA, Kashyap M, Pickering T, Dohadwala M (2025). Acute mitral block: pulse field ablation plus radiofrequency ablation when compared to radiofrequency ablation plus ethanol injection of vein of Marshall. *Journal of Interventional Cardiac Electrophysiology*.

[68] Valderrábano M, Peterson LE, Swarup V, Schurmann PA, Makkar A, Doshi RN (2020). Effect of Catheter Ablation With Vein of Marshall Ethanol Infusion vs Catheter Ablation Alone on Persistent Atrial Fibrillation: The VENUS Randomized Clinical Trial. *JAMA*.

[69] Derval N, Duchateau J, Denis A, Ramirez FD, Mahida S, André C (2021). Marshall bundle elimination, Pulmonary vein isolation, and Line completion for ANatomical ablation of persistent atrial fibrillation (Marshall-PLAN): Prospective, single-center study. *Heart Rhythm*.

[70] Scheinman MM, Morady F, Hess DS, Gonzalez R (1982). Catheter-induced ablation of the atrioventricular junction to control refractory supraventricular arrhythmias. *JAMA*.

[71] Reddy VY, Gerstenfeld EP, Natale A, Whang W, Cuoco FA, Patel C (2023). Pulsed Field or Conventional Thermal Ablation for Paroxysmal Atrial Fibrillation. *The New England Journal of Medicine*.

[72] Turagam MK, Neuzil P, Schmidt B, Reichlin T, Neven K, Metzner A (2023). Safety and Effectiveness of Pulsed Field Ablation to Treat Atrial Fibrillation: One-Year Outcomes From the MANIFEST-PF Registry. *Circulation*.

[73] Reichlin T, Kueffer T, Badertscher P, Jüni P, Knecht S, Thalmann G (2025). Pulsed Field or Cryoballoon Ablation for Paroxysmal Atrial Fibrillation. *The New England Journal of Medicine*.

[74] Ezzeddine FM, Asirvatham SJ, Nguyen DT (2024). Pulsed Field Ablation: A Comprehensive Update. *Journal of Clinical Medicine*.

[75] Anić A, Phlips T, Brešković T, Koopman P, Girouard S, Mediratta V (2023). Pulsed field ablation using focal contact force-sensing catheters for treatment of atrial fibrillation: acute and 90-day invasive remapping results. *Europace*.

[76] Ruwald MH, Johannessen A, Hansen ML, Haugdal M, Worck R, Hansen J (2024). Focal pulsed field ablation and ultrahigh-density mapping - versatile tools for all atrial arrhythmias? Initial procedural experiences. *Journal of Interventional Cardiac Electrophysiology*.

[77] Chun KRJ, Miklavčič D, Vlachos K, Bordignon S, Scherr D, Jais P (2024). State-of-the-art pulsed field ablation for cardiac arrhythmias: ongoing evolution and future perspective. *Europace*.

[78] Verma A, Asivatham SJ, Deneke T, Castellvi Q, Neal RE (2021). Primer on Pulsed Electrical Field Ablation: Understanding the Benefits and Limitations. *Circulation. Arrhythmia and Electrophysiology*.

[79] Sachdev M, Shanker A, Miller L, Smith A, Ellenbogen K, Chung M (2025). Biosense Webster Advisory: Update to the Instructions for Use for VARIPULSE. https://www.hrsonline.org/resource/biosense-webster-advisory-varipulse-instructions/.

[80] Nies M, Watanabe K, Kawamura I, Koruth JS (2024). Endocardial Pulsed Field Ablation and the Oesophagus: Are Atrio-oesophageal Fistulas Now History?. *Arrhythmia & Electrophysiology Review*.

[81] Tam MTK, Chan JYS, Chan CP, Wu EB, Lai A, Au ACK (2025). Effect of Pulsed-Field Ablation on Human Coronary Arteries: A Longitudinal Study With Intracoronary Imaging. *JACC. Clinical Electrophysiology*.

[82] Popa MA, Venier S, Menè R, Della Rocca DG, Sacher F, Derval N (2024). Characterization and Clinical Significance of Hemolysis After Pulsed Field Ablation for Atrial Fibrillation: Results of a Multicenter Analysis. *Circulation. Arrhythmia and Electrophysiology*.

[83] Caldwell J, Redfearn D (2012). Ablation of complex fractionated atrial electrograms in catheter ablation for AF; where have we been and where are we going?. *Current Cardiology Reviews*.

[84] Nademanee K, Lockwood E, Oketani N, Gidney B (2010). Catheter ablation of atrial fibrillation guided by complex fractionated atrial electrogram mapping of atrial fibrillation substrate. *Journal of Cardiology*.

[85] Verma A, Mantovan R, Macle L, De Martino G, Chen J, Morillo CA (2010). Substrate and Trigger Ablation for Reduction of Atrial Fibrillation (STAR AF): a randomized, multicentre, international trial. *European Heart Journal*.

[86] Ariyaratnam JP, Middeldorp ME, Brooks AG, Thomas G, Kadhim K, Mahajan R (2025). Coronary Sinus Isolation for High-Burden Atrial Fibrillation: A Randomized Clinical Trial. *JACC. Clinical Electrophysiology*.

[87] Kistler PM, Chieng D, Sugumar H, Ling LH, Segan L, Azzopardi S (2023). Effect of Catheter Ablation Using Pulmonary Vein Isolation With vs Without Posterior Left Atrial Wall Isolation on Atrial Arrhythmia Recurrence in Patients With Persistent Atrial Fibrillation: The CAPLA Randomized Clinical Trial. *JAMA*.

[88] Verma A STrategies for Catheter Ablation of peRsistent Atrial Fibrlllation (STARAF3). https://clinicaltrials.gov/study/NCT04428944.

[89] Di Biase L, Burkhardt JD, Mohanty P, Mohanty S, Sanchez JE, Trivedi C (2016). Left Atrial Appendage Isolation in Patients With Longstanding Persistent AF Undergoing Catheter Ablation: BELIEF Trial. *Journal of the American College of Cardiology*.

[90] Marrouche NF, Wilber D, Hindricks G, Jais P, Akoum N, Marchlinski F (2014). Association of atrial tissue fibrosis identified by delayed enhancement MRI and atrial fibrillation catheter ablation: the DECAAF study. *JAMA*.

[91] Marrouche NF, Wazni O, McGann C, Greene T, Dean JM, Dagher L (2022). Effect of MRI-Guided Fibrosis Ablation vs Conventional Catheter Ablation on Atrial Arrhythmia Recurrence in Patients With Persistent Atrial Fibrillation: The DECAAF II Randomized Clinical Trial. *JAMA*.

[92] Huo Y, Gaspar T, Schönbauer R, Wójcik M, Fiedler L, Roithinger FX (2022). Low-Voltage Myocardium-Guided Ablation Trial of Persistent Atrial Fibrillation. *NEJM Evidence*.

[93] Masuda M, Sunaga A, Tanaka N, Watanabe T, Minamiguchi H, Egami Y (2025). Low-voltage-area ablation for persistent atrial fibrillation: a randomized controlled trial. *Nature Medicine*.

[94] Piorkowski C, Kronborg M, Hourdain J, Piorkowski J, Kirstein B, Neudeck S (2018). Endo-/Epicardial Catheter Ablation of Atrial Fibrillation: Feasibility, Outcome, and Insights Into Arrhythmia Mechanisms. *Circulation. Arrhythmia and Electrophysiology*.

[95] Doll N, Weimar T, Kosior DA, Bulava A, Mokracek A, Mönnig G (2023). Efficacy and safety of hybrid epicardial and endocardial ablation versus endocardial ablation in patients with persistent and longstanding persistent atrial fibrillation: a randomised, controlled trial. *eClinicalMedicine*.

[96] Silberbauer J (2023). Pulmonary vein isolation vs endo-epicardial linear ablation for persistent atrial fibrillation: a randomized multicentre trial - EPIC AF. https://www.hra.nhs.uk/planning-and-improving-research/application-summaries/research-summaries/epic-af-trial/.

[97] Sang C, Liu Q, Lai Y, Xia S, Jiang R, Li S (2025). Pulmonary Vein Isolation With Optimized Linear Ablation vs Pulmonary Vein Isolation Alone for Persistent AF: The PROMPT-AF Randomized Clinical Trial. *JAMA*.

[98] Latcu DG, Milanese S, Kingston A, Canepa S, Lerebours C, Gutrod S (2023). Novel mapping tool to identify drivers in persistent atrial fibrillation: first clinical experience. *Europace*.

[99] Deisenhofer I, Albenque JP, Busch S, Gitenay E, Mountantonakis SE, Roux A (2025). Artificial intelligence for individualized treatment of persistent atrial fibrillation: a randomized controlled trial. *Nature Medicine*.

